# MicroRNA‐146b‐5p overexpression attenuates premature ovarian failure in mice by inhibiting the Dab2ip/Ask1/p38‐Mapk pathway and γH2A.X phosphorylation

**DOI:** 10.1111/cpr.12954

**Published:** 2020-11-09

**Authors:** Te Liu, Jiajia Lin, Chuan Chen, Xiaoli Nie, Fangfang Dou, Jiulin Chen, Zhenxin Wang, Zhangbin Gong

**Affiliations:** ^1^ Shanghai Geriatric Institute of Chinese Medicine Shanghai University of Traditional Chinese Medicine Shanghai China; ^2^ Department of Pathology Yale University School of Medicine New Haven CT USA; ^3^ Department of Laboratory Medicine of Zhongshan Hospital and Institute of Biomedical Science Fudan University Shanghai China; ^4^ Department of Biochemistry College of Basic Medicine Shanghai University of Traditional Chinese Medicine Shanghai China

**Keywords:** DAB2IP/ASK1/p38 pathway, high‐fat and high‐sugar diet, miR‐146b‐5p (miR‐146), poly (lactic‐co‐glycolic acid) nanoparticles, premature ovarian failure, γH2A.X

## Abstract

**Objective:**

To examine the role of high‐fat and high‐sugar (HFHS) diet‐induced oxidative stress, which is a risk factor for various diseases, in premature ovarian failure (POF).

**Materials and methods:**

Ovarian granulosa cells (OGCs) were isolated from mice and cultured in medium supplemented with HFHS and poly (lactic‐co‐glycolic acid) (PLGA)‐cross‐linked miR‐146b‐5p nanoparticles (miR‐146@PLGA). RNA and protein expression levels were examined using quantitative real‐time polymerase chain reaction and Western blotting, respectively. HFHS diet‐induced POF model mice were administered miR‐146@PLGA.

**Results:**

The ovarian tissue of mice fed a HFHS diet exhibited the typical pathological characteristics of POF. HFHS supplementation induced oxidative stress injury in the mouse OGCs, activation of the Dab2ip/Ask1/p38‐Mapk signalling pathway and phosphorylation of γH2A.X in vitro and in vivo. The results of the luciferase reporter assay revealed that miR‐146 specifically downregulated p38‐Mapk14 expression. Meanwhile, co‐immunoprecipitation and Western blot analyses revealed that HFHS supplementation upregulated nuclear p38‐Mapk14 expression and consequently enhanced γH2A.X (Ser139) phosphorylation. The HFHS diet‐induced POF mouse model treated with miR‐146@PLGA exhibited downregulated p38‐Mapk14 expression in the OGCs, mitigated OGC ageing and alleviated the symptoms of POF.

**Conclusions:**

This study demonstrated that HFHS supplementation activates the Dab2ip/Ask1/p38‐Mapk signalling pathway and promotes γH2A.X phosphorylation by inhibiting the expression of endogenous miR‐146b‐5p, which results in OGC ageing and POF development.

## Graphical abstract

To examine the role of high‐fat and high‐sugar (HFHS) diet‐induced oxidative stress, which is a risk factor for various diseases, in premature ovarian failure (POF). Ovarian granulosa cells (OGCs) were isolated from mice and cultured in medium supplemented with HFHS and poly (lactic‐co‐glycolic acid) (PLGA)‐cross‐linked miR‐146 nanoparticles (miR‐146@PLGA). RNA and protein expression levels were examined using quantitative real‐time polymerase chain reaction and Western blotting, respectively. HFHS diet‐induced POF model mice were administered miR‐146@PLGA. The ovarian tissue of mice fed a HFHS diet exhibited the typical pathological characteristics of POF. HFHS supplementation induced oxidative stress injury in the mouse OGCs, activation of the Dab2ip/Ask1/p38‐Mapk signalling pathway and phosphorylation of γH2A.X in vitro and in vivo. The results of the luciferase reporter assay revealed that miR‐146 specifically downregulated p38‐Mapk14 expression. Meanwhile, co‐immunoprecipitation and Western blot analyses revealed that HFHS supplementation upregulated nuclear p38‐Mapk14 expression and consequently enhanced γH2A.X (Ser139) phosphorylation. The HFHS diet‐induced POF mouse model treated with miR‐146@PLGA exhibited downregulated p38‐Mapk14 expression in the OGCs, mitigated OGC aging and alleviated the symptoms of POF. This study demonstrated that HFHS supplementation activates the Dab2ip/Ask1/p38‐Mapk signalling pathway and promotes γH2A.X phosphorylation by inhibiting the expression of endogenous miR‐146, which results in OGC aging and POF development.

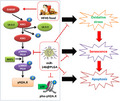

## 1 INTRODUCTION

Premature ovarian failure (POF) is characterized by amenorrhoea, low oestrogen, high gonadotropin and a lack of mature follicles in females aged below 40 years and leads to infertility.^1^, ^2^, ^3^, ^4^ The pathogenesis of POF is closely associated with the health and quality of ovarian granulosa cells (OGCs).^1^, ^5^, ^6^ Ageing and apoptosis of the OGCs contribute to a decline in ovarian reserve function.^5^, ^6^ Previously, we demonstrated that oxidative stress‐induced injury adversely affects OGC health.^1^ Recent studies have reported that a high‐fat and high‐sugar (HFHS) diet increases the risk of obesity, tumour formation, and cardiovascular disease^7^, ^8^, ^9^ and adversely affects ovarian function and ovum quality.^10^, ^11^, ^12^ However, further studies are needed to determine the pathogenetic mechanisms underlying the effects of HFHS diet‐induced obesity on OGC ageing and POF.

Differentially expressed in ovarian cancer 2/disabled homolog 2 (*DOC‐2*/*DAB2*), which is one of the two *fruitless* genes in Drosophila, is a phospholipid protein that is reported to function in the colony‐stimulating factor‐1 (CSF‐1) signal transduction pathway of macrophages.^13^, ^14^, ^15^ *DAB2* exhibits tumour suppressor activity and is associated with the development of various tumours.^13^, ^14^, ^16^ DOC2/DAB2 interaction protein (DAB2IP; also known as apoptosis signal‐regulating kinase 1‐interacting protein‐1 [AIP1]) directly interacts with DOC‐2/DAB2 to regulate various pathological characteristics of tumour cells, such as proliferation, invasion and apoptosis.^16^, ^17^, ^18^ Additionally, DAB2IP, which belongs to the Ras‐GTPase activating protein (RAS‐gap) family, exhibits tumour suppressor activity.^16^, ^17^ Protein kinase conserved domain 2 of DAB2IP can bind to apoptosis signal‐regulating kinase 1 (ASK1), phosphatase 2A (PP2A) and vascular endothelial growth factor receptor 2 (VEGFR2).^16^, ^17^ The *N*‐terminus of DAB2IP binds ASK1 and dephosphorylates it at Ser‐967. This results in activation of the p38‐MAPK14 and JNK pathways in the presence of tumour necrosis factor (TNF) through activation of the downstream TNFR1/TRADD/rip1/TRAF2 complex in endothelial cells, which leads to cell injury and apoptosis.^14^, ^15^, ^16^, ^17^ Activation of the DAB2IP/ASK1 signalling pathway is associated with cell ageing and death. On our previous studies, we found that high fat diet could induce development of mouse atherosclerosis by promoting the expression of the DAB2IP/ASK1 pathway in vascular endothelial cells. The results of molecular biology assay (immunofluorescence staining, qPCR and Western blotting) indicated that the expression levels of the mRNAs or proteins related to the DAB2IP/ASK1 pathway were significantly higher in the vascular endothelial cells of high fat diet treated group compared with control group. Meanwhile, the results of pathology assay showed that the plaque area, volume fraction of collagen fibres and lipid area in the aortic root was markedly elevated in the high fat diet treated group compared with the control group. Besides, the peripheral blood levels of total cholesterol, triglycerides and low‐density lipoprotein cholesterol were statistically significantly increased, whereas the high‐density lipoprotein cholesterol was statistically significantly decreased in the high fat diet treated group in comparison with the control group. Therefore, we have reason to believe that DAB2IP/ASK1 signal pathway is closely related to vascular injury and atherosclerosis induced by high‐fat diet. However, whether there is a close relationship between DAB2IP/ASK1 pathway and premature ovarian failure induced by high‐fat and high sugar diet has not been studied in depth.

MicroRNAs (miRs), a group of non‐coding RNAs with a length of 20‐23 nucleotides,^1^, ^2^, ^19^ do not encode proteins as they do not contain an open reading frame. The major function of miRs is downregulation of target gene expression by binding to the 3′‐untranslated coding region (3′ ‐UTR) of the target gene mRNA.^1^, ^2^, ^19^ miRs regulate many physiological and pathological processes, including the cell cycle, organ development, tumour development, neurodegenerative diseases, ageing and apoptosis.^1^, ^2^, ^19^ Previously, we demonstrated that miR‐15 negatively regulates the expression of *α‐Klotho* and *Lats1*, which leads to induction of mouse OGC death and POF.^1^, ^2^ This indicated that the development of POF is closely associated with the abnormal regulation of microRNAs.

In this study, the role of the Dab2ip/Ask1/p38‐Mapk pathway in mediating oxidative stress in OGCs and maintaining OGC health was examined using a HFHS diet‐induced POF mouse model. Furthermore, the role of miR‐146b‐5p (miR‐146) in regulating p38‐Mapk14 expression and γH2A.X phosphorylation, which promote ageing and apoptosis of the OGCs, was also examined in the model mice.

## 2 MATERIALS AND METHODS

A detailed description of all materials and methods can be found in Supporting Information (Materials and Methods).

### 2.1 Isolation and culture of mouse OGCs

Mouse OGCs were isolated and cultured according to the protocols in our previous study.^5^ Ten‐week‐old female C57BL/6 mice (n = 10) were purchased from the Experimental Animal Centre of Shanghai University of Traditional Chinese Medicine. The mice were sacrificed by cervical dislocation. Ovarian tissues were isolated under sterile conditions and incubated in ice‐cold (4°C) phosphate‐buffered saline (PBS). Next, the ovarian tissues were minced and digested with 2.0 mL of hyaluronidase (0.1%, Sigma‐Aldrich) for 1 min at 37°C. The digested sample was gently pipetted and incubated with 200 μL of foetal calf serum (Gibco) to terminate digestion. The suspension was then filtered through a 200‐mesh cell strainer. The filtrate was mixed with 5.0 mL of PBS and centrifuged at 300 *g* and 10°C for 5 minutes. The supernatant was discarded, and the pellet resuspended in 5.0 mL of PBS and centrifuged at 1500 rpm and 10°C for 5 minutes. The supernatant was discarded, and the cell pellet was resuspended in Dulbecco's Modified Eagle's Medium:Ham's F‐12 medium (1:1) supplemented with 10% foetal bovine serum, 10 ng/mL basic fibroblast growth factor, 10 ng/mL epidermal growth factor, 2 mmol/L l‐glutamine, 10 ng/mL growth hormone and 15 ng/mL estradiol (Gibco). The cell suspension was seeded in six‐well cell culture plates and cultured at 37°C in 5% CO_2_ until 80% confluency.

### 2.2 RNA extraction and quantitative real‐time polymerase chain reaction (qRT‐PCR)

Total RNA was extracted from the cells using TRIzol reagent (Invitrogen) according to the manufacturer's instructions. The residual genomic DNA was digested using DNase I (Sigma‐Aldrich). The extracted RNA was reverse transcribed into complementary DNA using the ReverTra Ace‐α First‐Strand cDNA synthesis kit (Toyobo). The qRT‐PCR analysis was performed using a RealPlex4 real‐time PCR detection system (Eppendorf Co. Ltd.) with SYBR Green Real‐time PCR Master Mix (Toyobo). The PCR cycling conditions were as follows: 40 cycles of 95°C for 15 seconds (denaturation), 58°C for 45 seconds (annealing) and 72°C for 42 seconds (elongation). The target gene threshold cycle (Ct) values were normalized using the following formulas:

ΔCt = Ct_genes - Ct_18SrRNAand

ΔΔCt =ΔCt_all_groups -ΔCt_blankcontrol_group;

where Ct_genes is the Ct value of the target gene, Ct_18SrRNA is the Ct value of the 18S rRNA gene, ΔCt_all_groups is the ΔCt value of the test group and ΔCt_blankcontrol_group is the ΔCt value of the control group.

The mRNA expression levels were normalized to those of 18S rRNA.

### 2.3 Co‐immunoprecipitation (Co‐IP) assay

Cells seeded in six‐well plates (3 × 10^5^/well) were cultured until 85% confluency. Next, the cells were lysed in a modified cell lysis buffer (500 μL/ plate; 20 mmol/L Tris [pH 7.5], 150 mmol/L NaCl, 1% Triton X‐100, 1 mmol/L EDTA, sodium pyrophosphate, β‐glycerophosphate, Na_3_VO_4_ and leupeptin; Beyotime Institute of Biotechnology). The samples were centrifuged to remove insoluble debris, and the supernatant was preincubated with 20 μg of protein A agarose beads (Beyotime) with rocking for 30 minutes at 4°C. Next, the samples were centrifuged and transferred to a fresh 1.5‐mL tube. The samples were incubated with the primary antibodies for 90 minutes, and then incubated with 20 μg of protein A agarose beads to capture the immune complexes. The pelleted beads were washed thrice with 500 μL of cell lysis buffer, dissolved in 4× sodium dodecyl sulphate‐polyacrylamide gel electrophoresis sample loading buffer and incubated at 95°C for 10 minutes.

### 2.4 **Loading of miR on poly (lactic‐co‐glycolic acid)** (**PLGA) nanomaterials**

Here, miR‐146 specifically refers to miR‐146b‐5p. The miR‐146b‐5p (miR‐146) and miR‐mut oligo RNAs were synthesized by Genepharma as reported previously.^20^, ^21^, ^22^ PLGA (MedChemExpress) was dissolved in methylene chloride overnight. miR‐146/miR‐mut was complexed with spermidine at an 8:1 polyamine nitrogen to nucleotide phosphate molar ratio. Next, 100 nmol/L of miR‐146/miR‐mut per 100 mg of polymer in Tris‐EDTA (10 mmol/L Tris‐HCl and 1 mmol/L EDTA) buffer (Sigma‐Aldrich) was added dropwise to the PLGA solution with agitation to obtain the first emulsion. To the sonicated first emulsion, 2.5% polyvinyl alcohol and 5 mg/mL avidin‐palmitate solution were added to obtain the second emulsion. The samples were stirred in 0.3% polyvinyl alcohol for 3 hours to evaporate the organic phase and harden the nanoparticles. To synthesize unmodified nanoparticles, the hardened nanoparticles were incubated in PBS without ligand for 30 minutes, while the second emulsion was prepared using only 2.5% polyvinyl alcohol. All nanoparticles were washed twice with deionized water to remove residual solvent, centrifuged at 4°C, lyophilized and stored at −20°C. As previously reported,^20^, ^21^, ^22^ 5 mg of miR‐PLGA was dissolved in 0.5 mL of methylene chloride for 30 minutes, and miR‐146/miR‐mut was extracted twice with Tris‐EDTA buffer. Encapsulation efficiency was determined by comparing the amount of miR loaded on the PLGA nanoparticles to the theoretical loading (1 nmol miR/mg polymer). To prepare the miR‐PLGA conjugate (nanoparticle‐miR‐CH2.5), 514 pmol of miR/mg of nanoparticle was loaded.

### 2.5 Induction of POF in mice by feeding a HFHS and injection of miR‐PLGA

Female C57BL/6 mice (n = 20) aged 10 weeks were purchased from the experimental animal centre of Shanghai University of Traditional Chinese Medicine.^7^, ^23^, ^24^ The mice were randomly divided into the following two groups (10 mice/group): the PLGA group, which was intravenously administered 400 μL of miR‐146@PLGA (20 mg/mL) through the caudal vena cava once every 3 days and the miR‐mut@PLGA group, which intravenously administered 400 μL of miR‐mut@PLGA (20 mg/mL) once every 3 days. The mice in the PLGA and miR‐mut@PLGA groups were fed a high‐fat diet (8 g/kg bodyweight) and administered 400 μL of 30% d‐glucose once a day via gavage for 30 days. The animal study was approved by the Ethics Committee of Shanghai Institute of Traditional Chinese Medicine, Geriatrics Department (SHIGESYDW2019019). All animal experiments were performed according to the experimental animal laws and regulations of the China National Science and Technology Commission.

### 2.6 Statistical analysis

At least three independent experiments were performed. Data are shown as mean ± standard error. The means were compared using Student's *t* test. Differences were considered significant at *P* < .05.

## 3 RESULTS

### 3.1 HFHS supplementation effectively induced oxidative stress injury in OGCs

**Figure 1:**
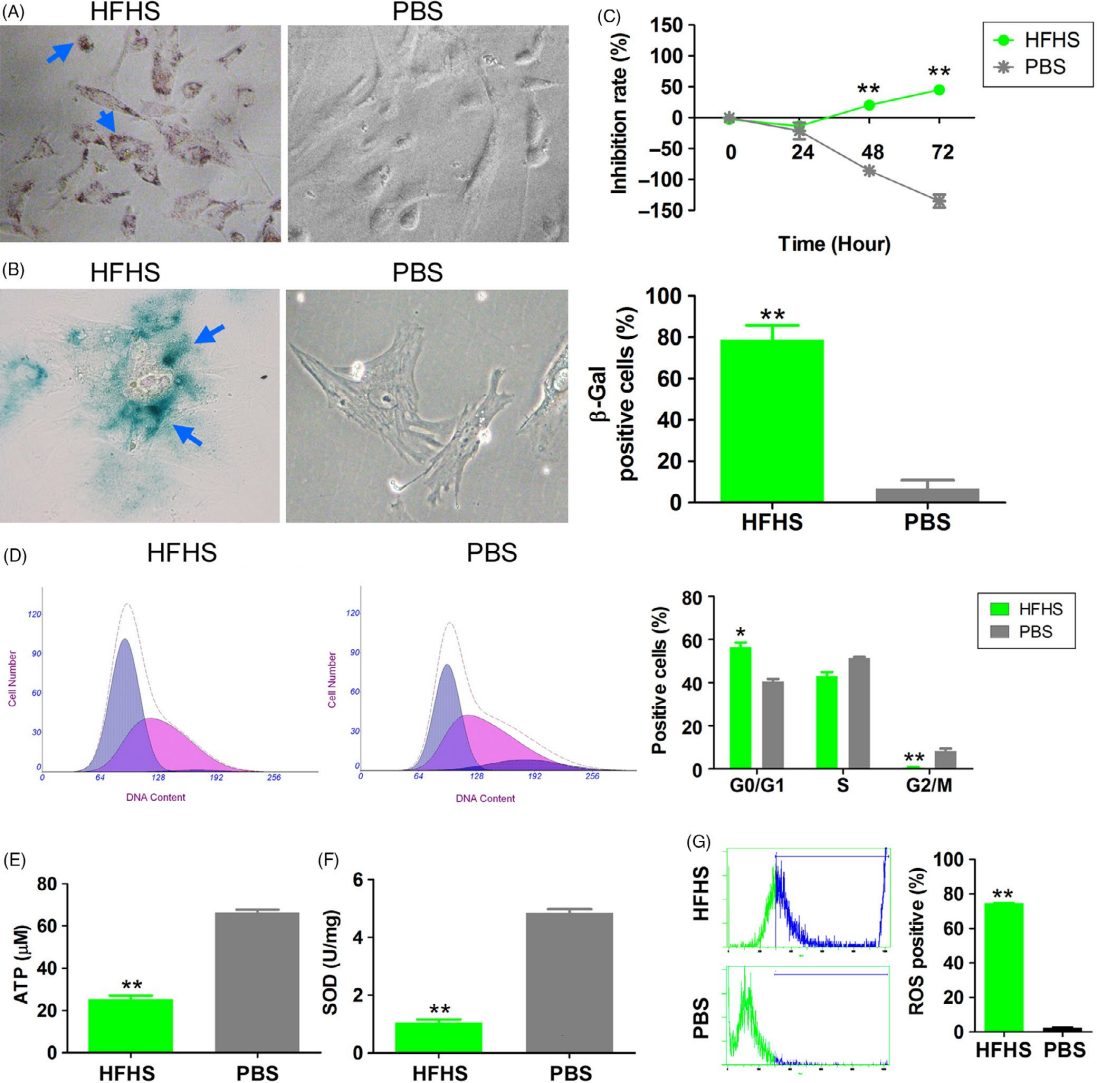
A high‐fat and high‐sugar (HFHS) diet effectively induces oxidative stress injury in ovarian granulosa cells (OGCs). A, Oil red O staining showed significantly red staining in the cytoplasm of cells in the HFHS group. Positive cells are indicated by arrows. Magnification 200×. B, β‐gal staining showed significantly red and blue staining in the cytoplasm of cells in the HFHS group. Positive cells are indicated by arrows. **P < .01 vs phosphate‐buffered saline (PBS) group, *t* test, n = 3. Magnification 200×. C, MTT results showed that the proliferation inhibition rate was significantly higher in the HFHS group than in the control group. **P < .01 vs PBS group, *t* test, n = 3. D, Flow cytometry showed that the proportion of cells in the G0/G1 phase increased significantly while those in the G2/M phase decreased in the HFHS group. **P < .01 vs PBS group, *P < .05 vs PBS group, *t* test, n = 3. E, ATP content was significantly lower in the HFHS group than in the vehicle group. **P < .01 vs PBS group, *t* test, n = 3. F, SOD activity was significantly lower in the HFHS group than in the vehicle group. **P < .01 vs PBS group, *t* test, n = 3. G, Reactive oxygen species (ROS) content was significantly higher in the HFHS group than in the vehicle group. **P < .01 vs PBS group, *t* test, n = 3

To examine the effect of a HFHS diet on OGCs, mouse OGCs cultured in medium supplemented with HFHS were treated with oxidized low‐density lipoprotein (oxLDL, 50 μg/mL) and d‐glucose (30 mmol/L) for 48 hours. The cells from the HFHS‐supplemented group exhibited significantly enhanced oil red O and β‐gal staining intensities in the cytoplasm, which indicated that the cells absorbed lipids and exhibited signs of ageing. In contrast, these changes were not observed in the PBS‐supplemented group (Figure 1A,B). The results of the (3‐(4,5‐dimethylthiazol‐2‐yl)‐2,5‐diphenyltetrazolium bromide (MTT) assay revealed that proliferation of the PBS‐supplemented group was time‐dependently higher than that of the HFHS‐supplemented group (Figure 1C). Flow cytometric analysis revealed that compared with the PBS‐supplemented group, in the HFHS group, there was a significantly higher proportion of cells in G0/G1 phase and a significantly lower proportion of cells in G2/M phase. This indicated that the cells were arrested at G0/G1 phase in the HFHS‐supplemented group (Figure 1D). Compared with the PBS‐supplemented group, adenosine triphosphate (ATP) content and superoxidase dismutase (SOD) activity were significantly lower and reactive oxygen species (ROS) was significantly higher in the HFHS‐supplemented group (Figure 1E‐G). These results indicated that HFHS supplementation could induce oxidative stress injury in OGCs, resulting in cell cycle arrest and growth inhibition.

### 3.2 HFHS supplementation activates the Dab2ip/Ask1/p38‐Mapk pathway and phosphorylates γH2A.X in mouse OGCs

**Figure 2:**
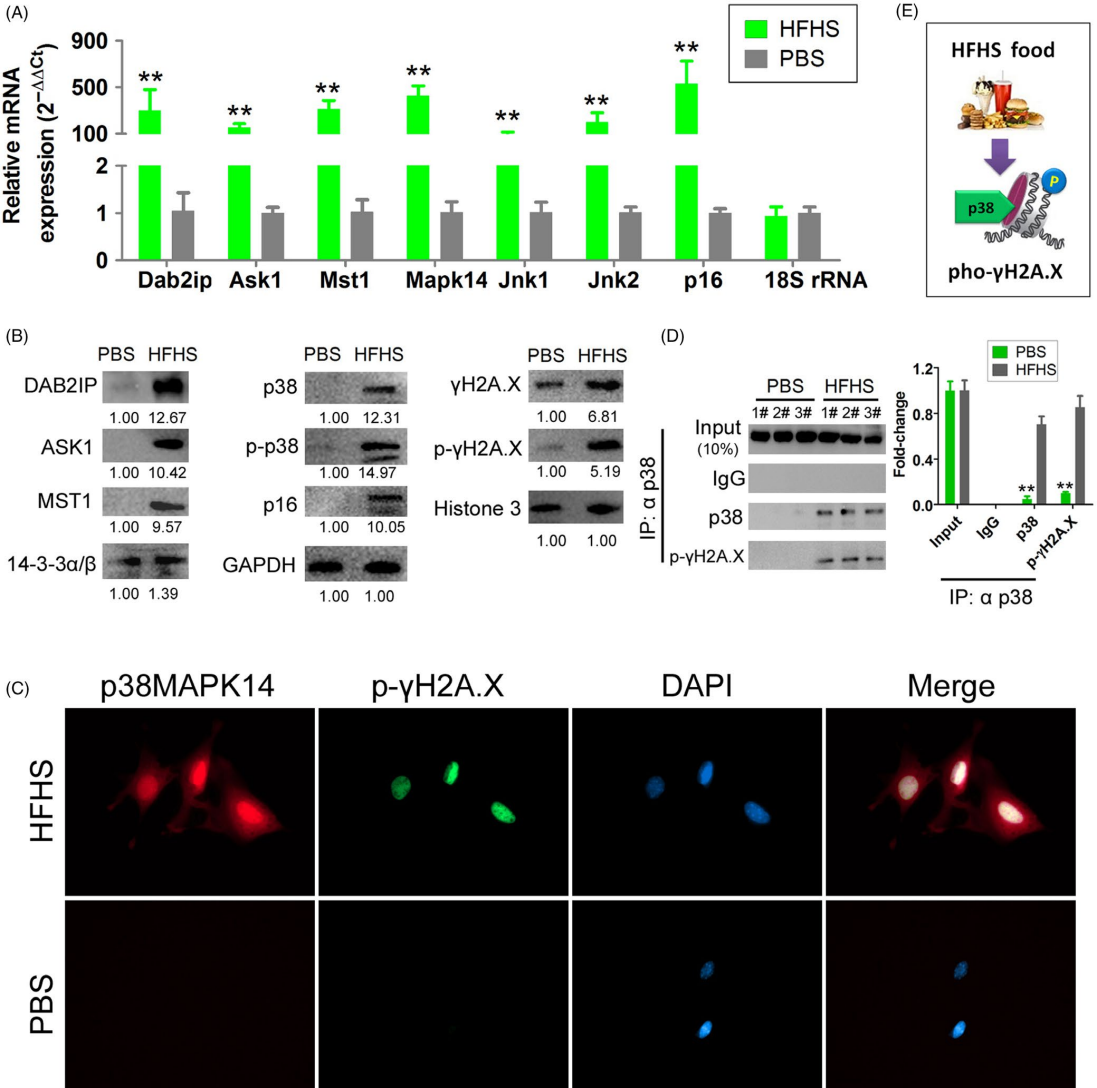
HFHS treatment activates the DAB2IP/ASK1/p38MAPK pathway and p‐γH2A.Xin mice OGCs. A, qRT‐PCR results show that the mRNA expression level of Dab2ip, Ask1, Mst1, p38Mapk14, Jnk1/2 and p16 was significantly higher in the OGCs of the HFHS group than in OGCs of the vehicle group. **P < .01 vs PBS group, *t* test, n = 3. B, Results of the Western blot analysis show the DAB2IP, ASK1, MST1, p38Mapk14, p16 and γH2A.X protein expression levels are higher in the HFHS group than in the vehicle group. C, The results of immunofluorescence staining indicate that HFHS could be stimulated high p38Mapk14 and p‐γH2A.X expression levels and coexpression in the nucleus. D, Results of the co‐immunoprecipitation (Co‐IP) and Western blot analysis showed a strong cross‐linking signal between the p38Mapk14 and p‐γH2A.X proteins in the OGCs of the HFHS group, which was very weak in that of the vehicle group. **P < .01 vs PBS group, *t* test, n = 3. E, Mechanism of p38Mapk14‐induced phosphorylation modification for γH2A.X

The qRT‐PCR analysis revealed that the mRNA expression levels of *Dab2ip*, *Ask1*, *Mst1*, *p38‐Mapk14*, *Jnk1/2* and *p16* in the HFHS‐supplemented group were significantly upregulated when compared with those in the PBS‐supplemented group (Figure 2A). Consistently, Western blotting analysis revealed that the expression levels of Dab2ip, Ask1, Mst1, p38‐Mapk14, p16 and γH2A.X in the HFHS‐supplemented group were significantly higher than those in the PBS‐supplemented group (Figure 2B). Meanwhile, the levels of phosphorylated p38‐Mapk14 (p‐p38‐Mapk14) and γH2A.X (p‐γH2A.X) in the HFHS‐supplemented group were higher than those in the PBS‐supplemented group (Figure 2B). Immunofluorescence analysis revealed that HFHS supplementation upregulated the expression and nuclear co‐localization of p‐p38‐Mapk14 and p‐γH2A.X (Figure 2C). Additionally, Co‐IP and Western blotting analyses revealed that the co‐localization of p‐p38‐Mapk14 and p‐γH2A.X was higher in the HFHS‐supplemented group than in the PBS‐supplemented group (Figure 2D). These results suggest that HFHS supplementation activates the Dab2ip/Ask1/p38‐Mapk signalling pathway in the mouse OGCs, which resulted in phosphorylation of γH2A.X (Figure 2E).

### 3.3 *p38‐Mapk14* is a target of miR‐146

**Figure 3:**
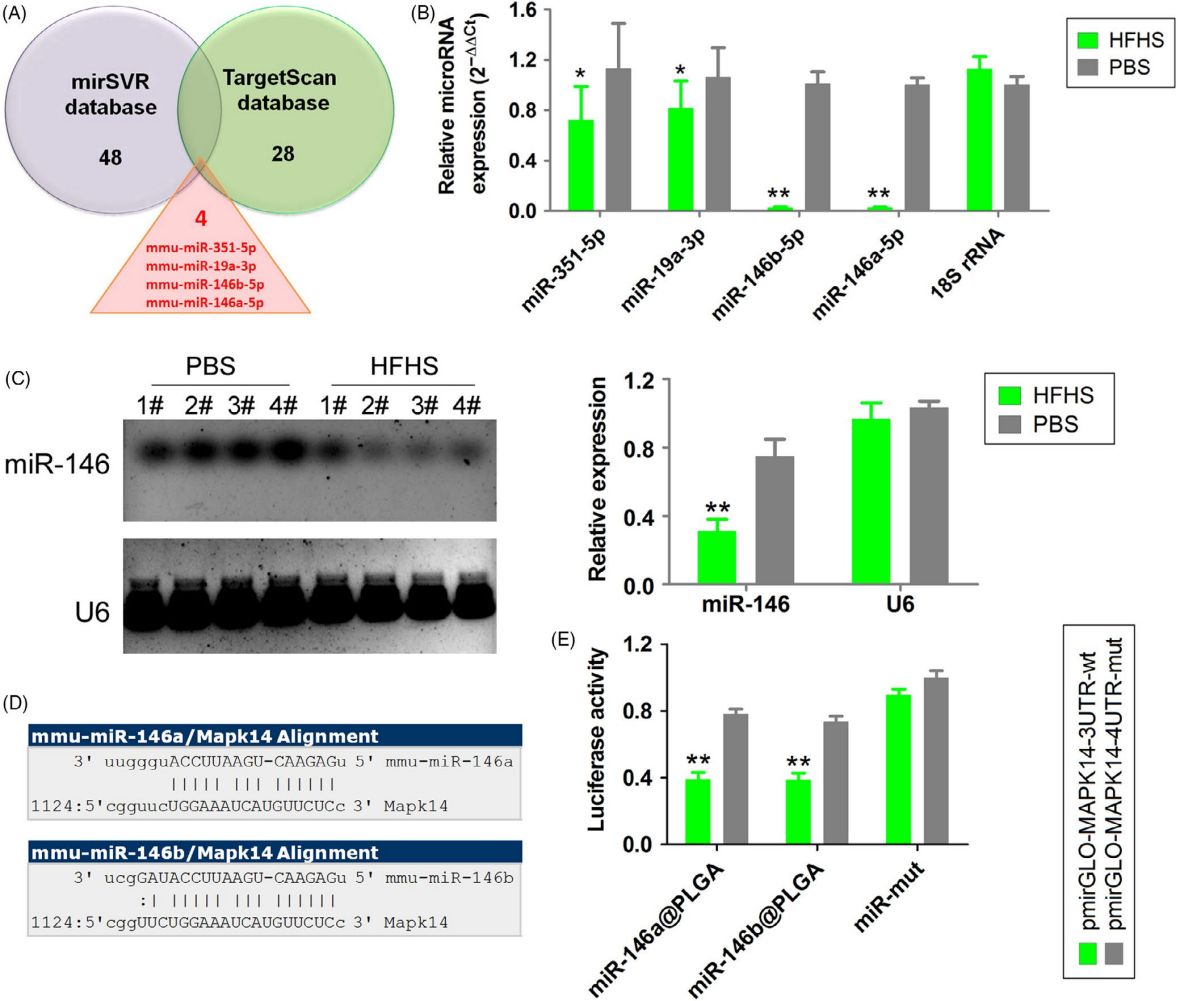
p38Mapk14 is one of the miR‐146‐specific targeting regulatory genes. A, Four common microRNAs were obtained between the mirSVR and TargetScan databases that have the potential in targeting regulatory p38 genes. B, qPCR results showed that miR‐146a‐5p and miR‐146b‐5p expression levels were significantly lower in HFHS‐treated OGCs than in control OGCs. **P < .01 vs PBS group, *P < .05 vs PBS group, *t* test, n = 3. C, Northern blot analysis also showed that the miR‐146 probe hybridization signal strength was significantly lower in the HFHS group than in the vehicle group. **P < .01 vs PBS group, *t* test, n = 4. D, Both miR‐146a‐5p and miR‐146b‐5p have six nucleotides that exhibit complementary pairing with the 3′‐UTR of p38Mapk14. E, Luciferase reporter assay results showed that both miR‐146a‐5p and miR‐146b‐5p could significantly reduce the site‐specific luciferase activity on the 3′‐UTR of the wild‐type p38Mapk14 gene

Analysis of the mirSVR (http://www.microrna.org/microrna/home.do) and TargetScan (http://www.targetscan.org) databases revealed 48 and 28 miRs that potentially target p38, respectively. Four miRs, miR‐351‐5p, miR‐19a‐3p, miR‐146a‐5p and miR‐146b‐5p, were detected in both databases (Figure 3A). The expression levels of miR‐146a‐5p and miR‐146b‐5p were significantly lower in the HFHS‐supplemented group than in the PBS‐supplemented group (Figure 3B). Northern blotting analysis revealed that the miR‐146b‐5p (miR‐146) signal was significantly weaker in the HFHS‐supplemented group than in the PBS‐supplemented group (Figure 3C). A sequence alignment revealed that both miR‐146a‐5p and miR‐146b‐5p have six nucleotides (5′‐GAGAAC‐3′) that are complementary to the 3′‐UTR of *p38‐Mapk14* mRNA (Figure 3D). The results of a luciferase reporter assay revealed that both miR‐146a‐5p and miR‐146b‐5p significantly decreased the luciferase activity of a construct containing the wild‐type *p38‐Mapk14* mRNA 3′‐UTR (Figure 3E). These results indicate that *p38‐Mapk14* is a target gene of miR‐146 (Here, miR‐146 specifically refers to miR‐146b‐5p).

### 3.4 miR‐146 overexpression significantly mitigated the HFHS‐induced oxidative stress injury in OGCs

**Figure 4:**
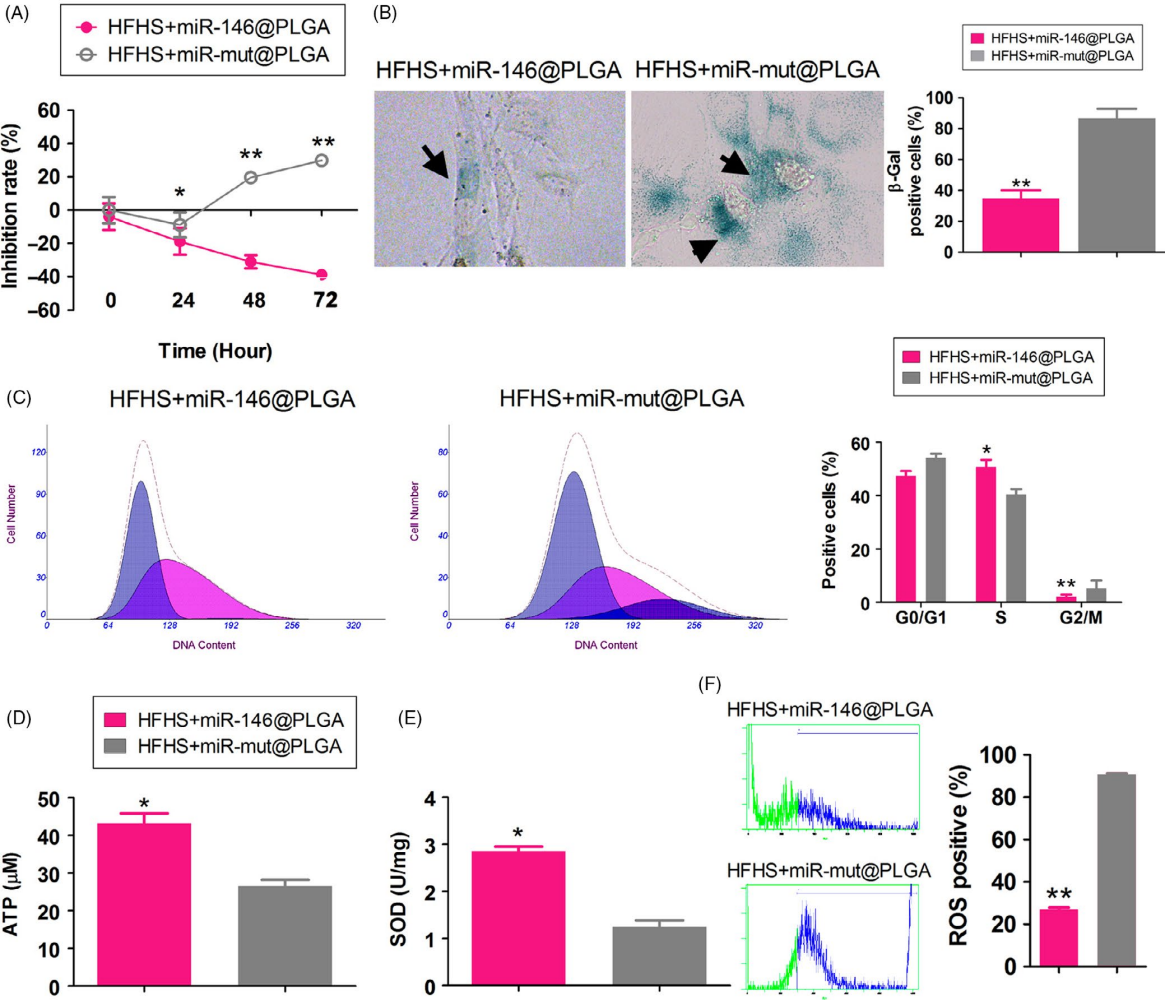
miR‐146 overexpression could significantly counteract the HFHS‐induced oxidative stress injury of OGCs. A, MTT results showed that the proliferation inhibition rate of OGCs was significantly lower in the HFHS + miR‐146@PLGA group than in the HFHS + miR‐mut@PLGA group. **P < .01 vs HFHS + miR‐mut@PLGA group, *P < .05 vs HFHS + miR‐mut@PLGA group, *t* test, n = 3. B, Results of the β‐gal staining showed that the proportion of blue‐positive cells in the HFHS + miR‐146@PLGA group was significantly lower than that in HFHS + miR‐mut@PLGA group. **P < .01 vs HFHS + miR‐mut@PLGA group, *t* test, n = 3. Magnification 200×. C, Flow cytometry results revealed a significantly higher proportion of S‐phase cells in the HFHS + miR‐146@PLGA group OGCs. **P < .01 vs HFHS + miR‐mut@PLGA group, *P < .05 vs HFHS + miR‐mut@PLGA group, *t* test, n = 3. D, ATP content was significantly higher in the HFHS + miR‐146@PLGA group than in the control group. *P < .05 vs HFHS + miR‐mut@PLGA group, *t* test, n = 3. E, SOD activity was significantly higher in HFHS + miR‐146@PLGA group OGCs compared with the control group. *P < .05 vs HFHS + miR‐mut@PLGA group, *t* test, n = 3. F, ROS content was significantly lower in HFHS + miR‐146@PLGA group OGCs compared with the control group. **P < .01 vs HFHS + miR‐mut@PLGA group, *t* test, n = 3

To study the effect of miR‐146 on cell function, mouse OGCs were harvested and randomly divided into the following two groups: miR‐146@PLGA, which was transfected with miR‐146@PLGA and miR‐mut@PLGA, which was transfected with miR‐mut@PLGA. The transfected cells were cultured in medium supplemented with HFHS. The results of the MTT assay revealed that the proliferation of the HFHS + miR‐146@PLGA group was time‐dependently higher than that of the HFHS + miR‐mut@PLGA group (Figure 4A). Furthermore, the proportion of β‐gal‐positive cells in the HFHS + miR‐146@PLGA group was significantly lower than that in the HFHS + miR‐mut@PLGA group (Figure 4B). Flow cytometric analysis revealed that the proportion of cells in S‐phase was significantly increased, while that in G2/M phase was significantly decreased in the HFHS + miR‐146@PLGA group (Figure 4C). Compared with cells in the HFHS + miR‐mut@PLGA group, ATP content and SOD activity were higher and ROS content was significantly lower in the HFHS + miR‐146@PLGA group (Figure 4D‐F). These results demonstrated that PLGA could successfully induce miR‐146 overexpression in the OGCs, which significantly alleviated HFHS‐mediated oxidative stress injury.

### 3.5 miR‐146 overexpression significantly inhibited the Dab2ip/Ask1/p38‐Mapk pathway and γH2A.X phosphorylation in HFHS‐supplemented mouse OGCs

**Figure 5:**
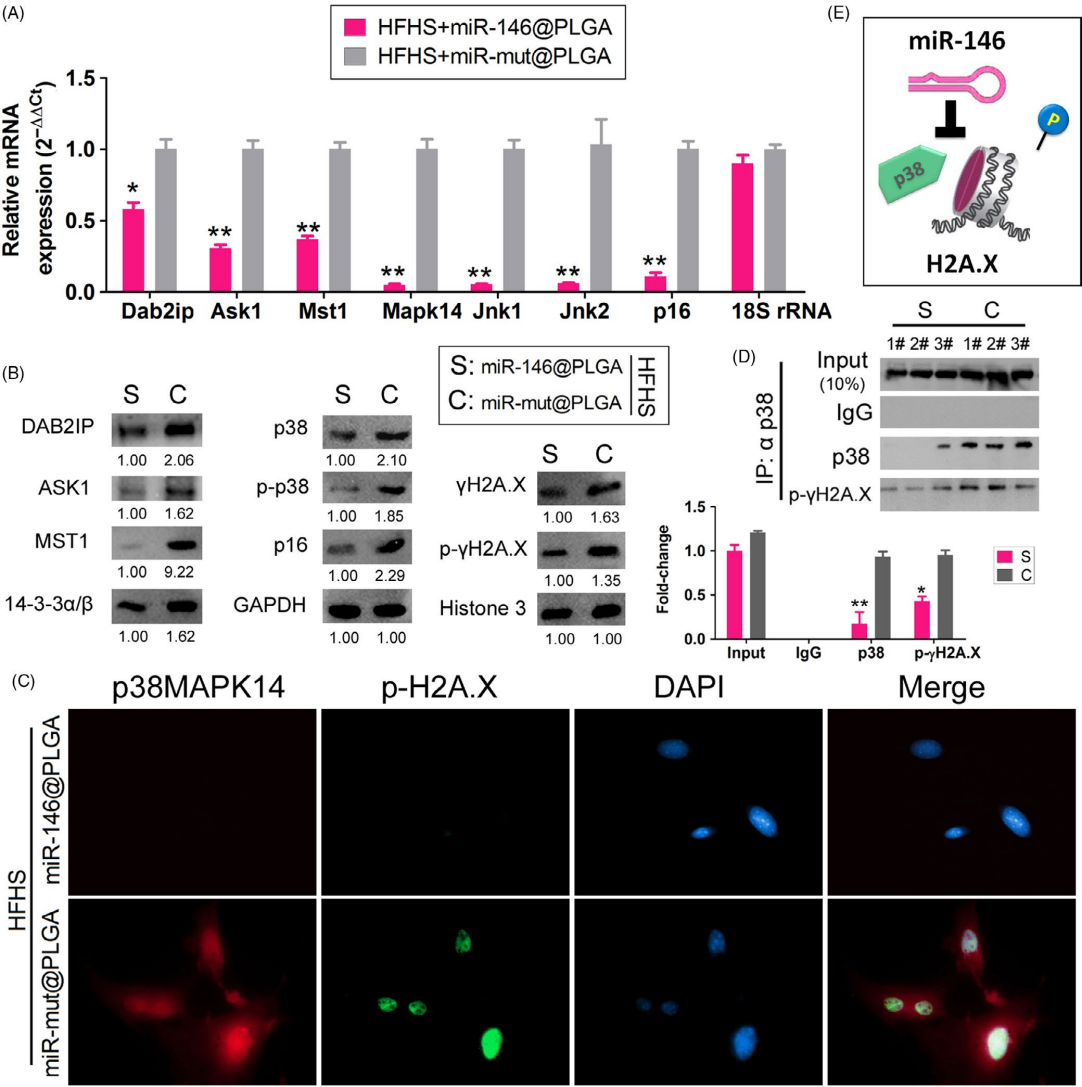
miR‐146 overexpression significantly weakens the HFHS‐induced activation of DAB2IP/ASK1/p38MAPK and p‐γH2A.X. A, qPCR results showed that Dab2ip, Ask1, Mst1, p38Mapk14, Jnk1/2 and p16mRNA expression levels were significantly downregulated in the inHFHS + miR‐146@PLGA group than in the HFHS + miR‐mut@PLGA group. **P < .01 vs HFHS + miR‐mut@PLGA group, *P < .05 vs HFHS + miR‐mut@PLGA group, *t* test, n = 3. B, Western blot results showed the protein expression of DAB2IP, ASK1, Mst1, p38mapk14, p16, and γH2A.X and p‐γH2A.X was significantly lower in the HFHS + miR‐146@PLGA group than in the control group. C, The results of immunofluorescence staining indicated that miR‐146@PLGA suppressed the expression of p38Mapk14 and p‐γH2A.X and coexpression in the nucleus. D, Co‐IP and Western blot results showed a much weaker cross‐linking signal between p38Mapk14 and p‐γ‐h2a.X protein in OGCs of the HFHS + miR‐146@PLGA group than in the HFHS + miR‐mut@PLGA group. **P < .01 vs HFHS + miR‐mut@PLGA group, *t* test, n = 3. E, The mechanism of miR‐146@PLGA suppressed phosphorylation modification of γH2A.X by p38Mapk14

The mRNA expression levels of *Dab2ip, Ask1, Mst1, p38‐Mapk14* *, Jnk1/2* and *p16* in the HFHS + miR‐146@PLGA group were downregulated when compared with the levels in the HFHS + miR‐mut@PLGA group (Figure 5A). Consistent with these findings, Western blotting analysis revealed that the protein expression levels of Dab2ip, Ask1, Mst1, p38‐Mapk14, p16 and γH2A.X in the HFHS + miR‐146@PLGA group were significantly lower than those in the HFHS + miR‐mut@PLGA group. Additionally, the levels of p‐p38‐Mapk14 and p‐γH2A.X were lower in the HFHS + miR‐146@PLGA group than in the HFHS + miR‐mut@PLGA group (Figure 5B). Immunofluorescence analysis revealed that miR‐146@PLGA suppressed the expression and nuclear co‐localization of p‐p38‐Mapk14 and p‐γH2A.X (Figure 5C). Co‐IP and Western blotting analyses revealed that the co‐localization of p38‐Mapk14 with p‐γH2A.X was weaker in the HFHS + miR‐146@PLGA group than in the HFHS + miR‐mut@PLGA group (Figure 5D). This suggested that miR‐146 overexpression significantly inhibited activation of the Dab2ip/Ask1/p38‐Mapk pathway and the phosphorylation of γH2A.X in HFHS‐supplemented mouse OGCs (Figure 5E).

### 3.6 miR‐146 overexpression alleviated HFHS diet‐induced POF in mice

**Figure 6:**
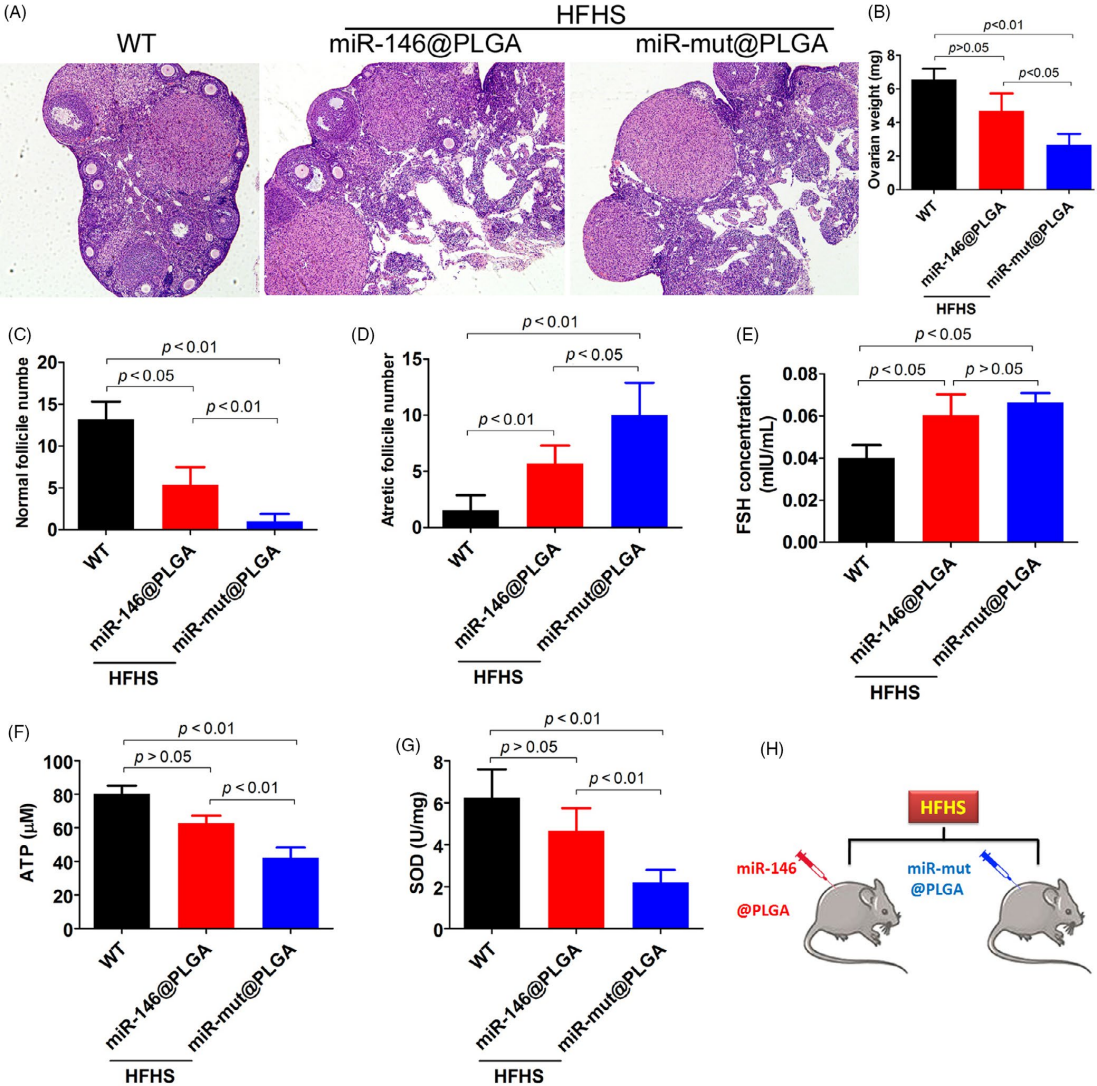
Overexpression of miR‐146 attenuates POF induced by HFHS in mice. A, Ovarian pathological identification was carried out by haematoxylin and eosin staining. Magnification 200×. B, Ovary weight was higher in the HFHS + miR‐146@PLGA group than in the HFHS + miR‐mut@PLGA group. C, Normal follicle count was higher in the HFHS + miR‐146@PLGA group than in the HFHS + miR‐mut@PLGA group. D, Fewer atretic follicles were present in HFHS + miR‐146@PLGA group mice than in HFHS + miR‐mut@PLGA group mice. E, The FSH level in the peripheral blood of HFHS + miR‐146@PLGAgroup mice was slightly lower than that in the peripheral blood of HFHS + miR‐mut@PLGA group mice. F, ATP content was significantly higher in the ovaries of HFHS + miR‐146@PLGA group mice than in the ovaries of HFHS + miR‐mut@PLGA group mice. G, SOD activity was significantly higher in the ovaries of HFHS + miR‐146@PLGAgroup mice than in the ovaries of HFHS + miR‐mut@PLGA group mice. H, Experimental diagram

**Figure 7:**
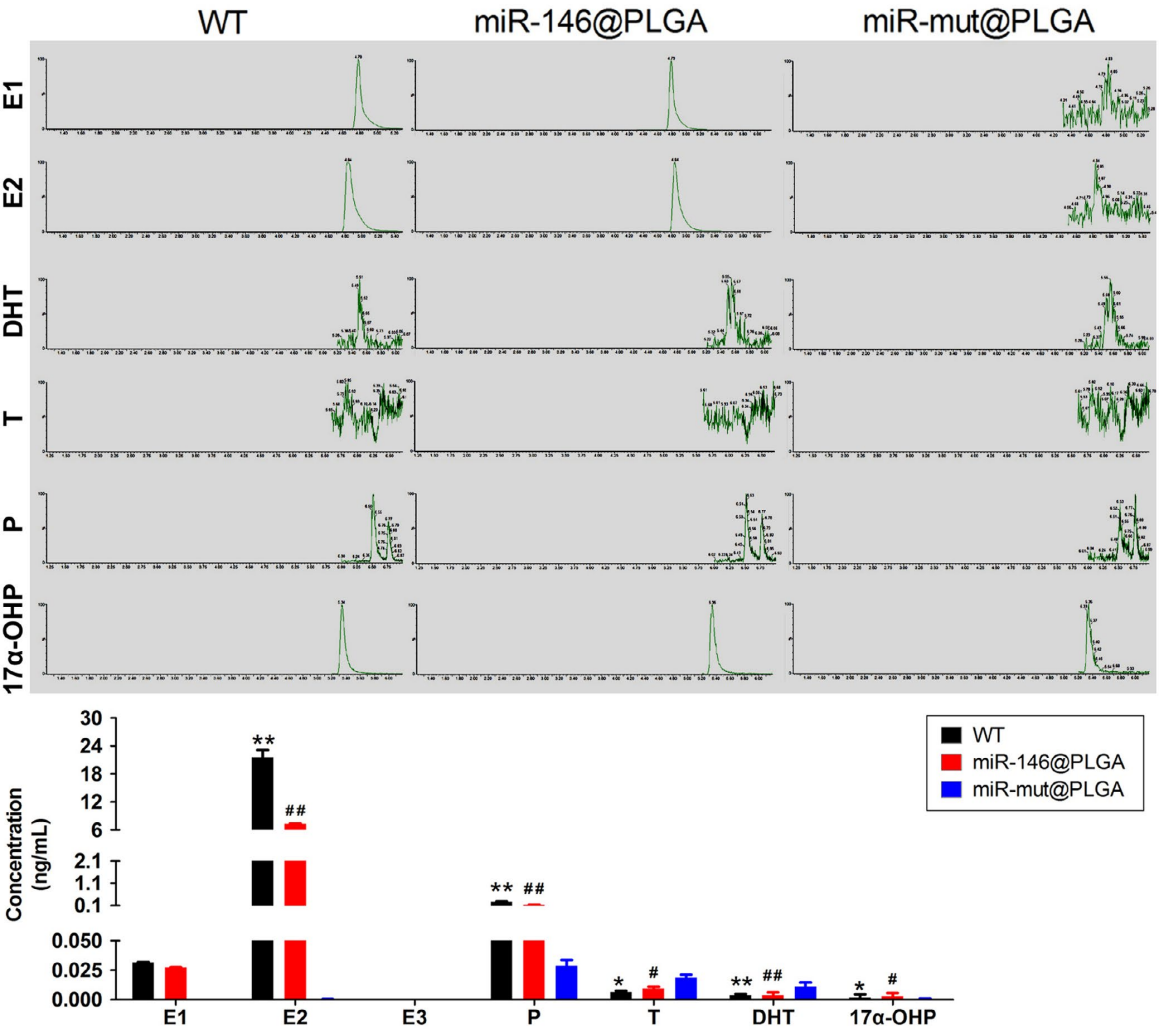
Mice steroid hormone level tested by HPLC‐MS/MS. E1, Estrone; E2, Estradiol; E3, Estriol; P, Progesterone; T, Testosterone; DHT, Dihydroxytestosterone; 17α‐OHP, 17α‐hydroxy pregnenolone. **P < .01 vs HFHS + miR‐mut@PLGA group, *P < .05 vs HFHS + miR‐mut@PLGA group, ^##^ *P* < .01 vs WT group, ^#^ *P* < .05 vs WT group, *t* test, n = 3

**Figure 8:**
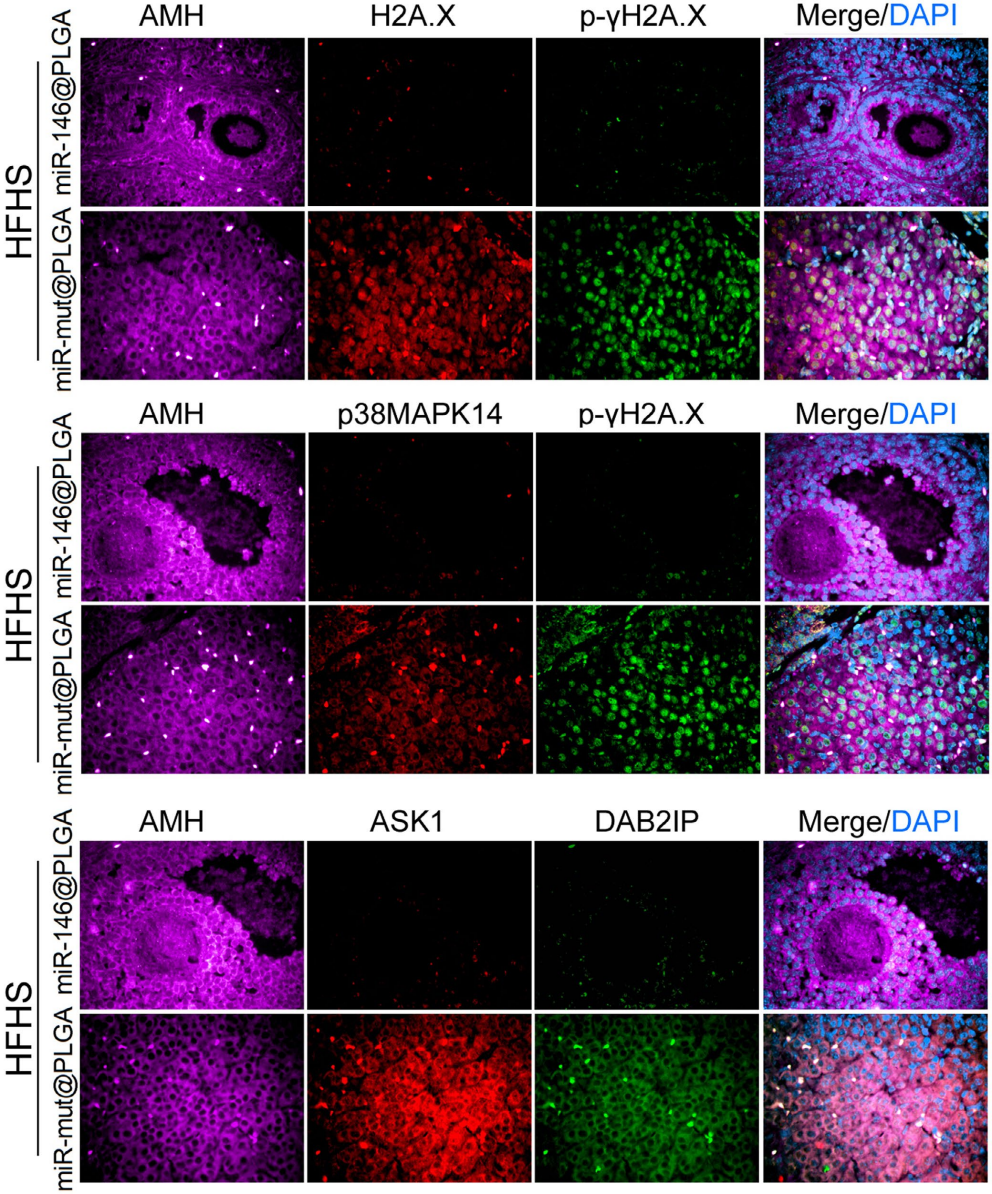
Results of ovarian immunofluorescence staining. Magnification 200×

To study the effect of miR‐146 overexpression on ovarian function, the mice were intraperitoneally injected with miR‐146@PLGA and fed a HFHS for 30 days. Ovary weight in the HFHS + miR‐146@PLGA group was higher than that in the HFHS + miR‐mut@PLGA group (Figure 6A,B). The HFHS + miR‐146@PLGA group exhibited a higher follicle count with fewer atretic follicles than the HFHS‐miR‐mut@PLGA group (Figure 6C,D). The follicle‐stimulating hormone levels in the peripheral blood of mice in the HFHS + miR‐146@PLGA group was non‐significantly lower than that of mice in the HFHS + miR‐mut@PLGA group (Figure 6E). ATP content and SOD activity were significantly higher in the HFHS + miR‐146@PLGA group than in the HFHS + miR‐mut@PLGA group (Figure 6F‐H). Steroid hormone levels were examined by high‐performance liquid chromatography‐tandem mass spectrometry. The peripheral blood levels of estradiol, progesterone and 17α‐hydroxy pregnenolone (17α‐OHP) in the HFHS + miR‐146@PLGA group were significantly higher than those in the HFHS + miR‐mut@PLGA group. In contrast, the peripheral blood levels of testosterone and dihydrotestosterone in the HFHS + miR‐146@PLGA group were significantly lower than those in the HFHS + miR‐mut@PLGA group (Figure 7). Immunofluorescence staining of OGCs (AMH‐positive cells) revealed that the H2A.X, p‐γH2A.X, p38‐Mapk14, Dab2ip and Ask1 signals in the HFHS + miR‐146@PLGA group were significantly weaker than those in the HFHS + miR‐mut@PLGA group (Figure 8). These results indicate that miR‐146 overexpression in mice could significantly mitigate HFHS‐induced oxidative stress injury and ageing in OGCs, inhibit Dab2ip/Ask1/p38‐Mapk activation and γH2A.X phosphorylation and consequently alleviate the symptoms of POF.

## 4 DISCUSSION

Several recent studies have focused on the adverse effects of a HFHS diet on health. Consumption of a HFHS diet can promote the development of various diseases, including cardiovascular disease, tumours and diabetes. Additionally, a HFHS diet has been recently reported to lead to ovarian dysfunction, which contributes to decreased female reproductive capacity. However, the mechanism underlying HFHS‐mediated ovarian dysfunction has not been elucidated. H2A.X, a histone protein, accounts for approximately 10% of the total H2A histones in human fibroblasts.^25^, ^26^, ^27^, ^28^, ^29^ Ionizing radiation, ultraviolet (UV) and ROS can activate H2A.X.^25^, ^26^, ^27^, ^28^, ^29^ Oxidative stress‐induced DNA damage promotes the phosphorylation of H2A.X at Ser139 by phosphoinositide 3 kinase‐like kinases, such as ATM, ATR and DNA‐PK,^25^, ^26^, ^27^, ^28^, ^29^ which results in activation of H2A.X. H2A.X accumulated at sites of DNA damage is phosphorylated at Ser139 within a few minutes of DNA damage. Phosphorylated H2A.X recruits various DNA damage‐associated proteins, such as MDC1, NBS1, RAD50, MRE11, 53BP1 and BRCA1.^25^, ^26^, ^27^, ^28^, ^29^ H2A.X has also been reported to be involved in the cellular response to DNA fragmentation. In response to different apoptosis signals, various kinases catalyse phosphorylation. Activation of the cell death receptor promotes the phosphorylation of H2A.X at Ser139 by DNA‐PK. Longwave UV radiation promotes the phosphorylation of H2A.X at Ser139 by c‐Jun amino‐terminal kinase (JNK1).^25^, ^26^, ^27^, ^28^, ^29^ Therefore, H2A.X activation is essential for checkpoint‐mediated cell cycle arrest and DNA repair after DNA double‐strand breaks.^25^, ^26^, ^27^, ^28^, ^29^ However, the role of HFHS diet‐induced oxidative damage in the phosphorylation and activation of H2A.X at specific sites has not been examined. Previous studies have reported that p38‐MAPK could catalyse the phosphorylation of H2A.X at Ser139 ^25^, ^26^, ^27^, ^28^, ^29^ under serum starvation conditions. This study explored the mechanism underlying HFHS diet‐induced cellular and organ damage involved oxidative stress.^7^, ^8^, ^9^ Oxidative stress was reported to promote the phosphorylation of H2A.X at Ser139. We hypothesized that phosphorylation of H2A.X at Ser139 may also occur during HFHS diet‐induced ageing and oxidative damage in OGCs.^25^, ^26^, ^27^, ^28^, ^29^ The findings of this study supported our hypothesis. Co‐IP revealed that the level of miR‐146, which negatively regulates p38‐Mapk14, was significantly downregulated during HFHS‐induced ageing of OGCs, which resulted in significant upregulation of p38‐Mapk14 expression. Activated p38‐MAPK enters the nucleus and activates H2A.X by phosphorylating it at Ser139. This study demonstrated, for the first time, the correlation of the DAB2IP/ASK1/p38 pathway with the phosphorylation and activation of γH2A.X. This enhanced our understanding of the mechanism underlying γH2A.X phosphorylation and revealed an intermediate ‘switch’ in the regulation of γH2A.X phosphorylation. The DAB2IP/ASK1/p38 pathway has been reported to be involved in oxidative stress‐induced vascular damage. However, DAB2IP/ASK1/p38 pathway‐catalysed γH2A.X phosphorylation has not been previously reported. Additionally, there are no studies on the correlation between miR‐146, the DAB2IP/ASK1/p38 pathway and γH2A.X phosphorylation in OGCs during POF. This study elucidated the mechanism underlying HFHS diet‐induced POF.

**Figure 9:**
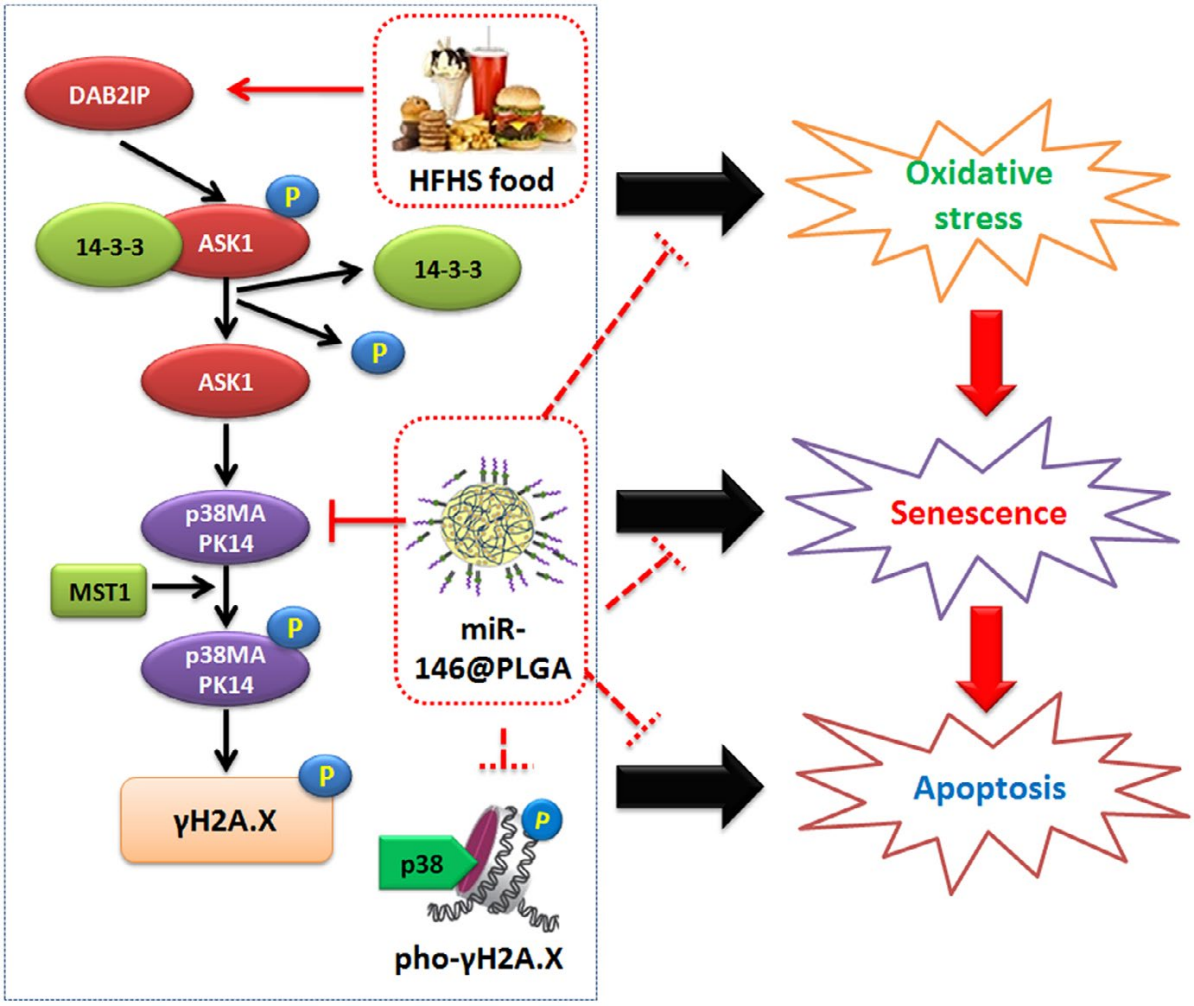
miR‐146@PLGA attenuates POF by suppressing activation of the DAB2IP/ASK1/p38 pathway and p‐γH2A.X

The novelty of this study included the use of PLGA nanoparticles as a carrier to successfully transfer and express miR‐146 in vivo and in vitro. PLGA is a degradable organic functional polymer, which comprises lactic acid and hydroxyacetic acid monomers. PLGA has applications in various industries, including the pharmaceutical and medical engineering industries, owing to its biocompatibility, non‐toxicity and good encapsulation and film formation performance.^23^, ^24^, ^30^ The degradation products of PLGA are lactic acid and hydroxyacetic acid, which are also by‐products of human metabolism. Thus, the application of PLGA for drug delivery or as a biomaterial does not result in toxic side effects (except in lactase‐deficient individuals).^23^, ^24^, ^30^ The monomer ratio can be adjusted and the degradation time for PLGA can be modulated as needed for various biomedical applications, such as skin transplantation, wound suture, in vivo implantation and generation of micro nanoparticles.^23^, ^24^, ^30^ In preclinical studies, PLGA has been used as a nanocarrier for therapeutics against cancer. Zhu et al used d‐α‐tocopherol polyethylene glycol succinate (TPGS) as a porogen. They prepared porous PLGA nanoparticles using a nanoprecipitation method and delivered docetaxel (DTX) and TPGS to HeLa cells, which were transplanted into in vivo tumour models. Studies on cytotoxicity and xenotransplantation tumour models revealed that the anti‐tumour effect of porous PLGA nanoparticles loaded with DTX/TPGS was higher than that of PLGA nanoparticles without TPGS. Additionally, porous PLGA nanoparticles loaded with DTX/TPGS could overcome multidrug resistance.^30^ Martin et al^20^ injected chitosan‐functionalized PLGA nanoparticles cross‐linked with an siRNA against GP130 (siGP130‐PLGA) into a bladder cancer xenograft mouse model, which resulted in significant downregulation of endogenous GP130 in vivo. Tumour volume in the siGP130‐PLGA group was decreased by approximately 70% when compared with that in the control group.^20^ Several studies have suggested that PLGA can be used as a carrier for protein, peptide and siRNA for delivery to cells. However, the ability of PLGA to deliver miRs into cells or organs has not been reported. Based on the findings of Martin et al,^20^ this study linked miR‐146 to chitosan‐functionalized PLGA nanoparticles to target p38‐Mapk14 in vitro and in vivo. PLGA could successfully deliver miR‐146 into OGCs in vitro and in vivo. The miR‐146@PLGA complex effectively downregulated the expression of p38‐Mapk14. Therefore, this study demonstrated that PLGA nanoparticles can carry and deliver miRs in vivo. Additionally, this study demonstrated that HFHS supplementation can activate the Dab2ip/Ask1/p38 signalling pathway and induce γH2A.X phosphorylation by inhibiting the expression of endogenous miR‐146 and promote OGC ageing, which resulted in the development of POF (Figure 9). Overexpression of miR‐146 using PLGA as a carrier mitigated p38‐induced γH2A.X phosphorylation, delayed OGC ageing and alleviated the symptoms of POF.

## CONFLICT OF INTEREST

We declared no potential conflicts of interest.

## AUTHORS' CONTRIBUTIONS

Te Liu, Jiajia Lin, Chuan Chen and Zhangbin Gong performed the majority of the experiments in the study. Xiaoli Nie, Fangfang Dou, Jiulin Chen and Zhenxin Wang contributed to the analysis of experimental data. Zhangbin Gong contributed to the study design, manuscript writing and provided experimental funding support. All authors read and approved the final manuscript.

## Supporting information

Supplementary Material

## Data Availability

The data sets used or analysed during the current study are available from the corresponding author on reasonable request.
