# Supplementary material for: MicroRNA‐146b‐5p overexpression attenuates premature ovarian failure in mice by inhibiting the Dab2ip/Ask1/p38‐Mapk pathway and γH2A.X phosphorylation
Source: Cell Prolif. 2020 Nov 9;54(1):e12954. doi: 10.1111/cpr.12954 (PMC7791167; doi:10.1111/cpr.12954)
Supplement: Supplementary file 1 — Supplementary Material [file CPR-54-e12954-s001.docx]

**MicroRNA-146b-5p overexpression attenuates premature ovarian failure in mice by inhibiting the Dab2ip/Ask1/p38-Mapk pathway and γH2A.X phosphorylation**

**Materials and methods**

**Isolation and culture of mouse OGCs**

According to our previous study [[1](#_ENREF_1)], ten-week-old female C57BL/6 mice (n = 10) were purchased from the Experimental Animal Centre of Shanghai University of Traditional Chinese Medicine. The mice were sacrificed by cervical dislocation.Ovarian tissues were isolated under sterile conditions and placed in 4°Cphosphate-buffered saline (PBS). The ovarian tissues were minced, and 2.0 mL of hyaluronidase (0.1%, Sigma-Aldrich, St Louis, MO, USA) was added for 1 min of digestion at 37°C. The tissue suspension was gently pipetted, and digestion was terminated by adding 200 μL of fetal calf serum (Gibco, Gaithersburg, MD, USA) to the suspension.The suspension was then filtered through a 200-mesh cell strainer. Next, 5.0 mL of PBS was added to the filtrate and mixed well, followed by centrifugation at 1500 rpm for 5 min at 10°C. The supernatant was discarded, the pellet was resuspended in 5.0 mL of PBS, and centrifuged at 1500 rpm for 5 min at 10°C. The supernatant was discarded, and the cell pellet was resuspended in Dulbecco’s Modified Eagle’s Medium:Ham’s F-12 medium (DMEM:F12) (1:1) and mixed well; the mediumcontained 10% fetal bovine serum, 10ng/mL basic fibroblast growth factor, 10ng/mL epidermal growth factor, 2mM l-glutamine, 10ng/mL growth hormone, and 15ng/mL estradiol(Gibco, Gaithersburg, MD, USA). The cell suspension was seeded in six-well cell culture plates, and then incubated at 37°C with 5% CO_2_ until 80% confluency.

**Oil Red O staining**

Briefly,each cell group was gently rinsed twice with PBS and fixed with 4% paraformaldehyde for 30 min. The PBS solution was rinsed for 1 min, and treated with oil red O working fluid and heated in an oven at 60 °C for 20 min.The cells were then washed twice with distilled water for 1 min each. Subsequently, 60% isopropanol was separated, and the red intracellular lipid droplets were observed under the microscope. The results were analyzed by processing the images through Image J software. Intracellular lipid droplets were expressed by integrated optical density.

**Senescence-associated beta-galactosidase (SA-β-Gal) staining**

This experiment was conducted using a SA-β-Gal Staining Kit (Beyotime Biotechnology, Zhejiang, China) according to the manufacturer’s instructions [[1](#_ENREF_1)]. Briefly, cells cultured in a 24-well plate were used. The conditioned medium was discarded, and the cells were washed once with PBS (Gibco). Then, 1 mL of staining fixative solution was added to the cell suspension, which was then fixed for 15 min at room temperature. After the fixative solution was discarded, the cells were washed thrice with PBS, for 3 min each time. For the staining, 1 mL of stain(working solution) was added to each well,the wells were incubated at 37°C overnight, and thecells were observed under a microscope on the following day.

**MTT assay**

Briefly, cell concentration was adjusted to 2000 cells/mL, cells were seeded into 96-well cell culture plates.Then, 10 µL of MTT solution (Beyotime) was added to each well, and the plates were incubated at 37°C for 3 h. The optical density value was measured at 450 nm with a Synergy2microplate reader. The cell proliferation inhibition rate (%) was calculated as follows:(1 – OD value of the experimental cells – blank /OD value of thecontrol cells – blank) ×100%.

**Detection of cell cycle by flow cytometry**

Briefly, 5×10^4^/mL of cells wereharvested for flow cytometry.Cells were fixed for 48 h with 1 mL of 70% precooled ethanol, then centrifuged at 1500 rpm for 5 min at 4°C. The cell pellet was then collected, stained with propidium iodide staining solution (Sigma Chemicals), then incubated in the dark at 4°C for 30 min. A flowcytometer (BD FACSAria) was used to analyze thecell cycle distribution of various cell groups. CellQuestsoftware was used for data analysis.

**ATP assay**

The ATP assay was performed using an enhanced ATP test kit (Beyotime), according to the manufacturer’s instructions. For the ATP assay, 1×10^5^cells/mL werelysed thoroughly with 200 µL of sample lysate, centrifuged at12000*g* for 5 min at 4°C.The supernatant was carefully collected. To prepare the ATP standard curve, the ATP standard was adjusted with a dilution buffer to final concentrations of 0.01, 0.03, 0.1, 0.3, 1, 3, and 10 μM, and this curve was used as a reference to determine the value of the sample. Working solutions for ATP detection were freshly made in accordance with the kit’s requirements. For the assay, 100 μL of working solution was added to the test and sample wells at the same time, and the plates were incubated at room temperature for 5 min. Then, 20 μL of sample or standard was added to the wells at the same time, quickly mixed, incubated at room temperature for 5 s, and the RLU value was measured on a luminometer.

**Reactive oxygen species (ROS) assay**

According to the instructions of the active oxygen test kit (Beyotime), briefly,2ʹ,7ʹ-dichlorodihydrofluorescein diacetate(DCFH-DA)was diluted with serum-free medium ata 1:1000 dilution to obtain a final concentration of 10 μM.Cells were digested with0.25%trypsin-EDTA, then centrifuged to collect the pellet, mixed with diluted DCFH-DA. The cellswere adjusted to 1×10^6^/mL, incubated at 37°C for 20 min, washed thrice with serum-free medium before flow cytometry(BD FACSAria)in the FL1 channel (excitation wavelength, 488nm; emission wavelength,525nm) for the detection of fluorescence in cells. Each sample was counted to have 1×10^5^ cells.

**SOD assay**

According to the instructions of the SOD activity test kit (Beyotime), briefly,1×10^5^/mLof cells werelysed thoroughlyusing with 200 µL ofsample lysate,centrifuged at12000*g* for 5 min at 4°C, and the supernatant wascollected. Then, fresh WST-8 enzyme working solutionwas prepared by mixing 151 µL of SOD detection buffer with 8 μL of WST-8 and 1 μL enzyme solution. At the same time, the SOD standard was diluted to 100, 50, 20, 10, 5, 2, 1U/mL to obtain a gradient. Then, 20 μL of the cell lysis supernatant or standard solution wasadded to 160 μL of fresh WST-8 enzyme working solution and 20 μL reaction starting solution, mixed well, and incubated at 37°C for 30 min. Absorbance was measured at 450 nm.

**RNA extraction and qRT-PCR**

Total RNA was extracted from each group of cells in accordance with the instructions ofTrizol Reagent (Invitrogen) and treated with Dnase I (Sigma-Aldrich) to remove residual genomic DNA. cDNA synthesis was carried out usingReverTra Ace-α First-Strand cDNA Synthesis Kit (Toyobo). qRT-PCR analysis was conducted using a RealPlex4 real-time PCR detection system (Eppendorf Co. Ltd., Germany), with SYBR Green real-time PCR Master Mix (Toyobo) as a fluorescent dye for nucleic acid amplification.The following reaction conditions were used for 40 cycles: denaturation at95°C for 15 s, annealing at 58°C for 45 s,and elongation at 72°C for 42s.For each sample, the maker gene Ct values were normalized with the following formula: ΔCt =Ct_genes–Ct_18sRNA, and ΔΔCt = ΔCt_all_groups–ΔCt_blankcontrol_group. The mRNAlevels were calibrated according to the 18S rRNA levels.

**Co-immunoprecipitation (Co-IP) assay**

Cells were seeded in 6-well plates at a density of 3×10^5^ per well and cultured until 85% confluent.The cells were then lysedin a modified cell lysis buffer (500μL per plate) for western blot and IPassays(20mM Tris [pH7.5], 150mM NaCl, 1% Triton X-100, 1mM EDTA, sodium pyrophosphate, β-glycerophosphate, Na_3_VO_4_, and leupeptin)(Beyotime). After lysis, each sample was centrifuged to clear the lysate of insoluble debris, and pre-incubated with 20μg of protein A agarose beads (Beyotime) with rocking for 30min at 4°C, centrifuged, and transferred to a fresh 1.5-mL tube. Primary antibodies were incubated for 90min before 20μg of protein A agarose beads were added again to capture the immune complexes. The pelleted beads were then washed thrice with 500μL of cell lysis buffer, dissolved in 4× sodium dodecyl sulfate-polyacrylamide gel electrophoresis (SDS-PAGE) sample loading buffer, and heated for 10min at 95°C.

**Western blot**

Briefly, total protein was extracted from cells in each group. Protein content was determined by using the BCA assay (Pierce Biotechnology, Inc., Rockford, IL, USA).Briefly, 20-μg protein samples were electrophoresed on 12% SDS-PAGE. Theseparatedproteins were transferred to a polyvinylidene difluoride (PVDF) membrane (Millipore, Billerica, MA,USA) for 45min at 37℃ after the blocking and membrane washing4 times with TBST for 1 mineach time. Each membrane was washed and incubated with the secondary antibodies for 45min. Immunoreactivity was visualized by performing an enhanced chemiluminescence (ECL) assay using an ECL kit from Perkin-Elmer LifeScience (Norwalk, USA).

**Northern blot analysis**

Northern blotting was performed as described previously[[2](#_ENREF_2)].Total RNA was extracted from the cells of all groups using the TRIzol extraction kit. After quantification, 20 μg of high-quality total RNA was used to performPAGE in 12% polyacrylamide (PAA) denaturing gel containing 7.5 M of urea. The electrophoresed RNAs were then transferred toa Hybond N^+^ nylon membrane (Amersham, Freiburg, Germany). The membrane was cross-linked under 1200 mJoule/cm^2^of UV lightfor 30 s and hybridized with the miR-146 antisense DNA probe todetect the expression of miR-146. After hybridization and membrane washing, the membrane was exposed to Kodak XAR-5 film for20–40 h (Sigma-Aldrich Chemical). Membranes that had been hybridized with the human U6 snRNA probe (5′-GCAGGGGCCATGCTAATCTTCTCTGTATCG-3′) were used as the positive control. The exposure time to the U6 snRNA probewas maintained at 15–30 min.

**Luciferase report assay**

A luciferase reporter assay was performed as previously described[[2](#_ENREF_2)]. Briefly, NIH-3T3 mouse embryonic fibroblast cells were seeded in 48-well plates at the concentration of 30,000 cells/well and cotransfected with 400 ng of miR-146a oligo RNA, miR-146b oligo RNA, or miR-mut oligo RNA (Genepharma, Shanghai, China) and 20 ng pmirGLO-MAPK14-3UTR-wt or pmirGLO-MAPK14-4UTR-mut (Novobiosci, Shanghai, China) using Lipofectamine-2000, according to the manufacturer’s protocol. Luciferase activity was measured after 48 h using the Dual-Luciferase Reporter Assay System (Promega, Madison, USA).

**HE staining**

Fresh tissue samples were collected and placed in 4% neutralbufferedparaformaldehyde(Sigma-Aldrich)for 30 min,and dehydrated using graded ethanol. Then, 6-μm-thick sections were cut from paraffin blocks and placedonto slides. Xylene(Sigma-Aldrich)was used for dewaxing. Tissue sections were stained with hematoxylin and eosin (H&E, Sigma-Aldrich),followed by clearing with xylene for 2 min, and sealing using neutral resin(Sigma-Aldrich).

**HPLC-MS/MS test**

Briefly, 250 μL of sample (calibration working solution or plasma) is mixed with 25μL of working solution and 250μL of methanol. The mixture is vortexed for 2 min. Then, 250μL of deionized water is added to the mixture and it is shaken for 1 min. The mixture was centrifugedfor 10000 *g* for 5 min, then transferred to an HLB elution plate, to which 600μL of extraction supernatant was added. The supernatant slowly flowed through the SPE plate under negative pressure and then into the waste drum. Acetonitrile/double distilled water (ddH_2_O; 1:9, v/v) was added to the SPE plate, followed by 200μL of *n*-hexane. The waste container is replaced by a 96-well plate. Then, 30μL methanol/acetonitrile (1:9, v/v) was added to the SPE plate to collect the filtrate. The filtrate was diluted with 50μLddH_2_O and swirled. It was then transferred to the wells of a 96-well plate and analyzed immediately by liquid chromatography-mass spectrometry/mass spectrometry (LC-MS/MS). Then, 10μL of the filtrate was injected into the water, and the temperature of the liquid mass analysis system and the automatic sampler wasset at 10°C. The binary mobile phase consisted of 0.3mm of NH_4_F (A) and methanol (B). The column temperature was kept at 35°C, and the mass spectrum parameters were as follows: capillary voltage of 3.56 kV, cone voltage of 50 V, source temperature of 150°C, desolvation temperature of 600°C, cone gas flow of 150 L/h, desolvation gas flow of 1000 L/h, and collision gas flow of 0.15 mL/min. Steroidal compounds were analyzed by electrospray ionization mass spectrometry (ESI-MS) and tandem MS in positive and negative ion modes. A total of 15 analytes were quantified by MRM, and 5 analytes (E1, E2, E3, Aldo, and 17-oh-pr) were quantified in negative ion mode.

**Immunofluorescence staining**

Briefly, all fresh tissues were immersed in 4% paraformaldehyde (Sigma-Aldrich) at room temperature for 30 min. Tissues were dehydrated using graded ethanol, embedded in paraffin, sectioned to 6-µm-thick slices, and soaked in xylene for dewaxing. The tissue sections were sealed at 37°C for 30 min with a solution (Beijing Biotechnology Co., Ltd., Zhejiang, China). The blocking solution was discarded, immunohistochemical cleaning solution (Beijing Biotechnology) was added, and the sections were washedthrice at room temperature for 5 min each. Then, the first antibody was added and incubate at 37°C for 45 min. The antibodieswere discarded and washed at room temperature for 5 mineach with of (Beyotime). Then, the secondary antibodies were added and incubate at 37°C for 45 min. The antibodies were discarded, and the sections were washed at room temperature for 5 min with (Beyotime). Finally, the immunofluorescence seal (Sigma-Aldrich) was added.

**Loading of microRNA on PLGAnanomaterials**

Briefly，miR-146 and miR-mut oligo RNAs were synthesized by Genepharma(Genepharma) as reported previously[[3-5](#_ENREF_3)]. Poly(lactic-co-glycolic acid) (PLGA; MedChemExpress, Shanghai, China)was dissolved overnight in methylene chloride, prior to formation of miR-146/miR-mut and spermidine complex at an 8:1 molar ratio of the polyamine nitrogen to the nucleotide phosphate. Then, 100 nM of miR-146/miR-mut per 100 mg of polymer in Tris-EDTA (10 mM Tris-HCl and 1 mM EDTA) buffer(Sigma-Aldrich) was added dropwise to the PLGA solution while vortexing. This solution was sonicated, and 2.5% polyvinyl alcohol and 5 mg/mL avidin palmitate solution were added to this for the second emulsion. The nanoparticles were hardened during solvent evaporation in 0.3% polyvinyl alcohol for 3 h. To synthesize unmodified nanoparticles, nanoparticles were incubated post-hardening in PBS without ligand for 30 min, and the second emulsion was made using only 2.5% polyvinyl alcohol. All nanoparticles were washed twice in deionized water to remove residual solvent, centrifuged at 4°C, lyophilized, and stored at –20 °C. As previously reported[[3-5](#_ENREF_3)],5 mg of miR-PLGA was dissolved in 0.5 mL of methylene chloride for 30 min, and miR-146/miR-mut was extracted twice into Tris-EDTA buffer. Encapsulation efficiency was determined by comparing the amount of microRNA loaded in PLGA nanoparticles with theoretical loading (1 nmol microRNA/mg polymer). For the miR-PLGA conjugate (nanoparticle-microRNA-CH2.5), the loading was 514 pmol microRNA/mg nanoparticle.

**Induction of POF by HFHS and microRNA-PLGAinjection in mice *in vivo***

Briefly, female C57BL/6 mice (n = 20) aged 10 weeks were purchased from experimental animal center of Shanghai University of traditional Chinese medicine referring to the reported methods [[6-8](#_ENREF_6)]. The mice were randomly divided into two groups of 10 each. Mice in the PLGA group receivedintravenous injections of 400μLmiR-146@PLGA (20mg/mL) through the vena caudalisonce every 3 days.The mice in the miR-mut@PLGAgroupreceived intravenous injections of 400μLmiR-mut@PLGA (20mg/mL) once every 3 days.All the 20 mice were fed a high-fat diet (8 g/kg) and treated with 400 μL of 30% d-glucose once a day via gavage for 30 days. The study was approved by the Ethics Committee of Shanghai Institute of Traditional Chinese Medicine,Geriatrics Department (SHIGESYDW2019019), and all the experiments were in line with the experimental animal laws and regulations of China National Science and Technology Commission.

**Statistical analysis**

Each experiment was performed at least thrice.Data were shown as the means ± standard error where applicable.Differences were evaluated with Student’s *t*test. A p value of<0.05 was considered statistically significant.

**Reference:**

1. Xiong Y, Liu T, Wang S, Chi H, Chen C, Zheng J. Cyclophosphamide promotes the proliferation inhibition of mouse ovarian granulosa cells and premature ovarian failure by activating the lncRNA-Meg3-p53-p66Shc pathway. Gene. 2017; 596: 1-8.

2. Cheng W, Liu T, Wan X, Gao Y, Wang H. MicroRNA-199a targets CD44 to suppress the tumorigenicity and multidrug resistance of ovarian cancer-initiating cells. FEBS J. 2012; 279: 2047-59.

3. Martin DT, Shen H, Steinbach-Rankins JM, Zhu X, Johnson KK, Syed J, et al. Glycoprotein-130 Expression Is Associated with Aggressive Bladder Cancer and Is a Potential Therapeutic Target. Mol Cancer Ther. 2019; 18: 413-20.

4. Martin DT, Steinbach JM, Liu J, Shimizu S, Kaimakliotis HZ, Wheeler MA, et al. Surface-modified nanoparticles enhance transurothelial penetration and delivery of survivin siRNA in treating bladder cancer. Mol Cancer Ther. 2014; 13: 71-81.

5. Woodrow KA, Cu Y, Booth CJ, Saucier-Sawyer JK, Wood MJ, Saltzman WM. Intravaginal gene silencing using biodegradable polymer nanoparticles densely loaded with small-interfering RNA. Nat Mater. 2009; 8: 526-33.

6. Goncalves MD, Lu C, Tutnauer J, Hartman TE, Hwang SK, Murphy CJ, et al. High-fructose corn syrup enhances intestinal tumor growth in mice. Science. 2019; 363: 1345-9.

7. Swider E, Maharjan S, Houkes K, van Riessen NK, Figdor C, Srinivas M, et al. Forster Resonance Energy Transfer-Based Stability Assessment of PLGA Nanoparticles in Vitro and in Vivo. ACS Appl Bio Mater. 2019; 2: 1131-40.

8. Cheng J, Teply BA, Sherifi I, Sung J, Luther G, Gu FX, et al. Formulation of functionalized PLGA-PEG nanoparticles for in vivo targeted drug delivery. Biomaterials. 2007; 28: 869-76.
